# The Influence of Conventional Treatment on Symptoms and Complaints in Patients With Chronic Postsurgical Hypoparathyroidism

**DOI:** 10.1002/jbm4.10586

**Published:** 2022-02-01

**Authors:** Bettina Stamm, Martina Blaschke, Lara Wilken, Deborah Wilde, Christina Heppner, Andreas Leha, Christoph Herrmann‐Lingen, Heide Siggelkow

**Affiliations:** ^1^ Endokrinologikum Saarbruecken Saarbruecken Germany; ^2^ Clinic of Gastroenterology, Gastrointestinal Oncology and Endocrinology University Medical Center Goettingen Goettingen Germany; ^3^ MVZ Endokrinologikum Goettingen Goettingen Germany; ^4^ Institute for Medical Statistics University Medical Center Goettingen Goettingen Germany; ^5^ Department for Psychosomatic Medicine and Psychotherapy University Medical Center Goettingen Goettingen Germany

**Keywords:** HYPOPARATHYROIDISM, QUALITY OF LIFE, SYMPTOMS AND COMPLAINTS, CONVENTIONAL THERAPY, ACTIVE VITAMIN D TREATMENT

## Abstract

Quality of life (QoL) is impaired in patients with chronic hypoparathyroidism (HypoPT). With a recently developed specific patient questionnaire, the 28‐item Hypoparathyroid Patient Questionnaire (HPQ 28), we were able to demonstrate an effect of laboratory parameters on symptoms and complaints identified by scales and items of the HPQ 28. Here, we evaluated the effect of conventional treatment modalities on QoL using this specific questionnaire. In this cross‐sectional study, we included 49 HypoPT (41 female and 8 male) patients. Laboratory values of total serum calcium, magnesium, phosphate, calcium‐phosphate product (CPP), and 24‐hour urine for calcium and phosphate were analyzed. Patients completed the HPQ 28 questionnaire during the corresponding visit. Mean age was 57.3 ± 10.5 years and duration of disease 12.6 ± 9.8 years. Most patients (86%, *n* = 42) were treated with the active vitamin D analogs calcitriol, alfacalcidol, or dihydrotachysterol (DHT). The use of calcium and magnesium supplements influenced scales on HPQ 28 in a dose‐dependent manner. We detected a dose‐dependent increase on the HPQ 28 scales “depression and anxiety” and “pain and cramps,” and the item “numbness and tingling” related to calcitriol. This effect was independent of gender, age, underlying disease, kind of surgery, serum 25‐hydroxyvitamin D_3_, calcium, or phosphate values. This study presents the first data on specific symptoms of HypoPT patients dependent on different treatment modalities. Our data suggest that in part the reduced QoL in these patients might be caused by conventional treatment. © 2022 The Authors. *JBMR Plus* published by Wiley Periodicals LLC on behalf of American Society for Bone and Mineral Research.

## Introduction

Chronic postsurgical hypoparathyroidism (HypoPT) is defined by hypocalcemia and inappropriately low levels of parathyroid hormone (PTH) lasting more than 6 months after the corresponding surgical procedure.^(^ ^1^ ^)^ The recommended treatment aims to maintain serum calcium levels within the lower normal range or even slightly below. Levels of phosphate and calcium‐phosphate product (CPP), magnesium, and 24‐hour urine values are supposed to lie within the gender‐specific reference ranges, and vitamin D status needs to be adequate.^(^ ^1^ ^)^ The treatment regimen is designed to free patients of the symptoms and signs of hypocalcemia. The primary therapy recommended is to prescribe activated vitamin D analogs in combination with calcium supplements, with additional measures required for patients in whom the target ranges are unattainable through this treatment. General vitamin D supplementation with 400 to 800 IU/d is part of this recommendation.^(^ ^1^, ^2^ ^)^ However, not all patients are free of symptoms, nor are their levels within the target ranges under the recommended treatment.

The management required to reach these goals tends to differ from country to country. In the United States, a higher amount of calcium and lower doses of activated vitamin D are used to keep phosphate levels down,^(^ ^3^ ^)^ in comparison to European studies.^(^ ^4^, ^5^, ^6^ ^)^ Moreover, not all of the activated vitamin D analogs on the global market are available in every country; eg, alfacalcidol is available in Europe^(^ ^4^, ^5^ ^)^ but not in the United States.^(^ ^3^ ^)^ As a result, there is currently no comparison of the different medications with respect to the target levels of laboratory parameters according to present guidelines^(^ ^1^, ^2^ ^)^ and the recommended treatment aims.

Together with recommendations on target levels of laboratory parameters, the European guidelines focused for the first time on the well‐being of patients and their quality of life (QoL).^(^ ^1^ ^)^ By now, a number of studies characterize the impaired QoL for patients with HypoPT.^(^ ^4^, ^5^, ^7^, ^8^ ^)^

Siggelkow and colleagues^(^ ^9^ ^)^ described interference with the activities of daily life and personal relationships in addition to cognitive, emotional, and physical symptoms. Meanwhile, the influence on work productivity and working ability is also part of the studies.^(^ ^4^, ^9^ ^)^ Comparing HypoPT with the QoL associated with other chronic diseases such as diabetes or heart failure^(^ ^9^ ^)^ has underlined the importance of this aspect to the treatment of HypoPT, which is now internationally acknowledged.^(^ ^10^, ^11^ ^)^

A number of HypoPT‐specific instruments to assess symptoms relating to QoL have been developed lately.^(^ ^12^, ^13^, ^14^ ^)^ We also recently developed a disease‐specific hypoparathyroid patient questionnaire, the 28‐item Hypoparathyroid Patient Questionnaire (HPQ 28), to be used in the daily care of HypoPT patients.^(^ ^14^ ^)^ This questionnaire enabled us to demonstrate an effect of the laboratory values on QoL when compared to two control groups.^(^ ^15^ ^)^ Here we analyze the effect of different treatment modalities on laboratory values and symptoms using the HPQ 28 in patients with chronic postoperative HypoPT.

## Patients and Methods

### Study design

We conducted a cross‐sectional study in two different endocrinologic centers (Goettingen and Saarbruecken) in Germany. Forty‐nine HypoPT patients were prospectively enrolled in the study as and when they attended the clinic. Patients received information on the study during their regular clinical checkup visits. After providing informed consent, they completed the HPQ 28 during their visit to one of the centers. During their routine control visit, different serum and urinary parameters were analyzed. The study was approved by the Ethics Review Board of University Medical Center Goettingen (No. 25/10/15); all the subjects provided written informed consent prior to participation and the data were pseudonymized. All the questionnaires were in German.

### Patients

We recruited patients determined as suffering from chronic postsurgical HypoPT, which was defined by the presence of hypocalcemia, inappropriately low PTH levels, and patients needing treatment at least 6 months after thyroid surgery.

HypoPT patients were excluded if their HypoPT proved to be idiopathic, genetic, or transient (less than 6 months diagnosed with HypoPT), they were under 18 or over 85 years of age, pregnant, unable to understand and answer the questionnaires, or were suffering from polyglandular autoimmune syndrome.

### Laboratory values

Laboratory values were determined immediately after blood sampling using standard laboratory methods (total serum calcium, serum albumin, serum magnesium, serum phosphate, and 25‐OH‐vitamin D3). Calcium adapted for albumin and CPP were calculated values from the laboratory (amedes MVZ wagnerstibbe, Goettingen). Twenty‐four‐hour urine collections were analyzed for calcium, creatinine, and phosphate with standard laboratory methods.

### Questionnaires

The HPQ 28 is a questionnaire we designed on the basis of previous testing to measure the symptoms and complaints typical in HypoPT patients.^(^ ^14^ ^)^ The results of initial application in a HypoPT study group have been described.^(^ ^15^ ^)^ The questionnaire comprises five different scales and three single items. The scales represent identified complaints in HypoPT: pain and cramps (PaC); loss of vitality (Vit); gastrointestinal symptoms (GiS); depression and anxiety (DaA); and neurovegetative complaints (NVS). The single items are numbness and tingling in certain parts of the body (numbness and tingling), memory difficulties, and heart palpitations.^(^ ^14^ ^)^

### Statistics

Data were analyzed with IBM‐SPSS software version 26 (IBM Corp., Armonk, NY, USA). Descriptive data were compared using chi‐squared or Fisher's exact test for categorical variables. Answers to items on the HPQ 28 were coded as follows: 0 = not at all; 1 = slightly; 2 = moderately; and 3 = severely. Group differences were evaluated using either one‐way analysis of variance (ANOVA) for normally distributed continuous values or the nonparametric Kruskal‐Wallis test (with included Dunn‐Bonferroni test in SPSS) for non‐normally distributed data. Data are presented as means ± standard deviation (SD) or standard error of the mean (SEM) for each of the five scales or single items. A pairwise complete case analysis was performed for few missing data (4/245 items in PaC scale were missing). For correlation analyses, Spearman's rank correlation was chosen for non‐normally distributed values, whereas Pearson correlations were performed for normally distributed values. For linear regression the adjusted *r*‐square (*r*_c_^2^) values were given for the goodness of fit, as well as the *p* values for the significance of linearity and the standardized coefficient parameter β. Bonferroni correction for the multiple comparison to the eight scales and items in the HPQ 28 was applied.

## Results

### Patient characteristics and laboratory parameters

The main characteristics of the 49 HypoPT patients (*n* = 35 Goettingen, *n* = 14 Saarbruecken) participating in this trial have been described.^(^ ^15^ ^)^ Patients were 57.3 ± 10.5 years old and were predominantly female (84%). The duration of disease in patients was 12.6 ± 9.8 years. In 43% of the cases, goiter was the underlying disease, followed by carcinoma (29%) and Graves' disease (12%). Most of the patients (84%) underwent a total thyroidectomy. Patient characteristics relating to the different treatment modalities are depicted in Table 1.

**Table 1:** Patient Characteristics Relating to the Different Treatment Modalities

Parameter	No active vitamin D_3_ (*n* = 7)	Calcitriol (*n* = 14)	Alfacalcidol (*n* = 21)	DHT (*n* = 6)	Calcitriol+ Alfacalcidol (*n* = 1)^a^	*p*
Age (years), mean ± SD (range)	55 ± 9 (44–69)	56 ± 10 (41–77)	59 ± 12 (41–75)	56 ± 6 (48–65)	74	0.70^b^
Gender (male/female)	1/6	3/11	2/19	1/5	1/0	0.74^c^
BMI (kg/m^2^), mean ± SD	33.0 ± 4.7	29.4 ± 4.4	27.4 ± 9.7	30.5 ± 9.1	30.1	0.50^b^
Calcium intake (yes/no)	2/5	10/4	15/6	4/2	0	0.2^c^
Calcium intake^d^ (mg/d), mean ± SD	800 ± 282	1240 ± 1003	885 ± 745	700 ± 383		0.3^b^
Magnesium intake (yes/no)	1/6	3/11	4/17	2/4	0	0.8^c^
Magnesium intake^d^ (mg/d)	300	540 ± 393	218 ± 96	250 ± 71		0.7^b^
Native vitamin D (yes/no)	4/3	7/7	12/9	2/4	0	0.4^c^
Native vitamin D intake^d^ (IU/d), mean ± SD	5657 ± 9,571	3520 ± 2,941	9590 ± 12,547	571 ± 606		0.50^b^
Type of surgery						0.047^c^
Total	2	8	8	5		1.0^a^
Subtotal	0	2	1	1	1	
Near total	0	1	0	0		
Not classified^a^	5	3	12	0		
Underlying disease						0.47^c^
Carcinoma	1	5	5	2	1	
Goiter	3	5	12	1		
Graves' disease	1	2	2	1		
Nodules	1	1	1	1		
pHPT	0	1	1	0		
Other	1	1	0	1		

BMI = body mass index; pHPT = primary hyperparathyroidism; SD = standard deviation.

^a^ Not included in statistical analysis.

^c^ Fisher's exact test.

^b^ Kruskal‐Wallis group analysis.

^d^ All the patients included recorded as taking the respective supplement.

The laboratory values were well adjusted as recommended in the guidelines from 2015.^(^ ^1^ ^)^ In summary, serum calcium/albumin values (*n* = 46) were between 1.6 and 2.39 mmol/L (mean 2.09 mmol/L; reference values = 2.0–2.6 mmol/L), serum phosphate values (*n* = 48) 0.84–1.75 mmol/L (mean 1.26 mmol/L; reference values = 0.8–1.6 mmol/L), CPP (*n* = 47) 1.87–3.63 mmol^2^/L^2^ (mean 2.71 mmol^2^/L^2^; reference values ≤4.4 mmol^2^/L^2^), 25OH vitamin D3 (*n* = 48) 13–176 nmol/L (mean 101 nmol/L; reference values = 72.5–139 nmol/L), serum magnesium (*n* = 39) 0.60–0.86 mmol/L (mean 0.78 mmol/L; reference values = 0.66–1.07 mmol/L), urinary 24‐hour calcium excretion (*n* = 25) 0.49–10.09 mmol/d (mean 5.39 mmol/d; reference values = 2.5–7.5 mmol/d), and urinary phosphate excretion (*n* = 21) 13.24–35.52 mmol/d (mean 21.76 mmol/d; reference values = 19.37–50.5 mmol/d).

### HypoPT medication

In general, the symptoms HypoPT patients present and report might not only be influenced by the disease itself, but also by the kind of treatment as well as the dose to reach and maintain target ranges according to the guidelines.^(^ ^1^ ^)^ Table 1 presents patients' characteristics and the different medications used in the study group (*n* = 49).

The majority of HypoPT patients (65%) received some preparation of calcium supplementation, either solely as calcium carbonate, (*n* = 24 patients) or calcium carbonate in combination with native vitamin D (Calcimagon® [500 mg Ca + 800 IU Vitamin D], *n* = 6; Calcimed® [500 mg Ca + 1000 IU Vitamin D], *n* = 1; Calcigen® [600 mg Ca + 400 IU vitamin D], *n* = 1). Magnesium supplements were a component of the medication in 10 patients (20%). At the time point of the study, two patients received teriparatide 1–34. Most patients (86%, *n* = 42) were treated with the active vitamin D analogs calcitriol, alfacalcidol, or dihydrotachysterol (DHT). Half of the patients were treated with alfacalcidol (52%, *n* = 22). One patient was treated with a combination of calcitriol and alfacalcidol. In part, the treatment concept in Saarbruecken aimed at creating and maintaining predominantly high levels of 25‐OH‐vitamin D3 as the central pillar, adding active vitamin D forms as needed. Thus, patients treated in Saarbruecken (*n* = 7) received a mean dose of 17,960 IU/d of cholecalciferol. Five patients were treated with 20,000–40,000 IU/d. The mean dose was higher than in patients treated with cholecalciferol in Goettingen (*n* = 14, mean dose = 2209 IU/d, only one patient was treated with 10,000 IU/d). Values for 25‐hydroxyvitamin D_3_ correlated significantly with the dose of genuine vitamin D_3_ (*p* < 0.001; *r* = 0.512, data not shown). Seven patients were not treated with active vitamin D analogues, but with either high doses of genuine vitamin D_3_, or calcium alone, or magnesium alone, or calcium as part of their normal diet or calcium‐rich mineral water instead.

Patients supplementing calcium (according to either regimen) mostly took 500 mg/d (*n* = 9) or 1000 mg/d (*n* = 10). The dose of magnesium was 300 mg/d in 40% of patients. Most patients treated with native vitamin D received between 1000 and 2857 IU per day (*n* = 14). Seven patients received thiazides, mostly 25 mg/d (*n* = 5). A detailed table of treatments and doses is provided in Appendix S1, Table A.

### The influence of active vitamin D treatment on laboratory values

**Fig 1:**
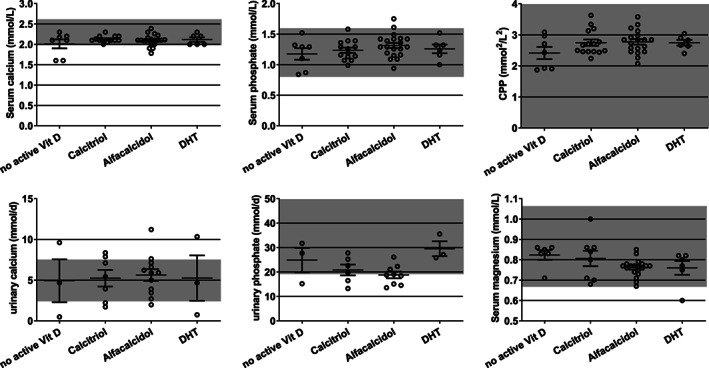
Calcium, phosphate, CPP, and magnesium serum and calcium, phosphate urine treated with active vitamin D compounds calcitriol, alfacalcidol, or DHT. Reference ranges are marked in gray. CPP = calcium‐phosphate product; DHT = dihydrotachysterol.

To investigate whether the laboratory values for serum calcium, serum phosphate, serum magnesium, CPP, as well as the urinary calcium and urinary phosphate values were dependent on different active vitamin D compounds, we compared HypoPT patients with respect to the active vitamin D form taken (Fig. 1).

The serum values of all patients treated with calcitriol lay within the reference range (Table 2), whereas some patients treated with alfacalcidol (serum calcium, serum phosphate) or treated with DHT (serum magnesium) did not. More patients treated with alfacalcidol had values within the urinary calcium reference range, whereas more patients reached the reference range for urinary phosphate when treated with calcitriol or DHT.

**Table 2:** Percentage of Patients Within the Reference Ranges for Serum and Urinary Parameters

Parameter	No intake % (*n*)	Calcitriol % (*n*)	Alfacalcidol % (*n*)	DHT % (*n*)
Serum calcium (2.0–2.6 mmol/L)	71 (*n* = 7)	100 (*n* = 14)	84 (*n* = 19)	100 (*n* = 6)
Serum phosphate (0.8–1.6 mmol/L)	100 (*n* = 7)	100 (*n* = 15)	90 (*n* = 20)	100 (*n* = 6)
CPP (<4.4 mmol^2^/L^2^)	100 (*n* = 7)	100 (*n* = 15)	100 (*n* = 19)	100 (*n* = 6)
Urinary calcium excretion (2.5–7.5 mmol/d)	33 (*n* = 3)	43 (*n* = 7)	75 (*n* = 12)	33 (*n* = 3)
Urinary phosphate excretion (19.37–50.5 mmol/d)	66 (*n* = 3)	66 (*n* = 6)	44 (*n* = 9)	100 (*n* = 3)
Serum magnesium (0.66–1.07 mmol/L)	100 (*n* = 6)	100 (*n* = 8)	100 (*n* = 19)	83 (*n* = 6)

CPP = calcium‐phosphate product; DHT = dihydrotachysterol; *n* = number of patients.

We found no significant differences between the different treatments with respect to serum or urinary parameters (Fig. 1). Pearson correlation analysis between calcitriol or alfacalcidol intake and laboratory parameters revealed no significant correlation to any parameter.

### The influence of active vitamin D treatment on scales and items of the HPQ 28

**Fig 2:**
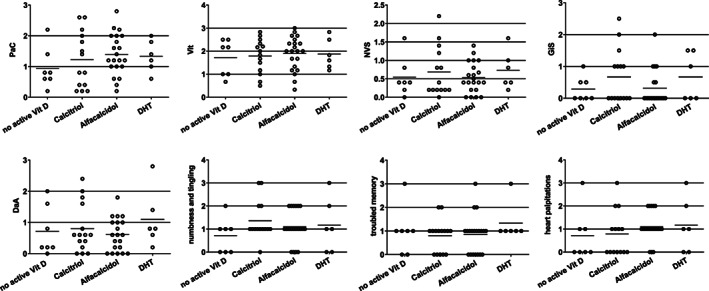
Score levels of scales and items of the HPQ 28 with reference to the active Vit‐D compound in the HypoPT patient group (*n* = 49); PaC and Vit (six items; 4/294 items in 49 patients missing); GiS (two items; 1/98 items in 49 patients missing); DaA (five items; 1/245 items in 49 patients missing); NVS (five items; 4/245 items in 49 patients missing); one patient did not answer to item numbness and tingling and one did not answer to troubled memory. DaA = depression and anxiety; DHT = dihydrotachysterol; GiS = gastrointestinal symptoms; NVS = neurovegetative symptoms; PaC = pain and cramps; Vit = loss of vitality.

To evaluate whether symptoms according to HPQ 28 were dependent on different active vitamin D compounds although laboratory serum values did not differ (Fig. 1), we compared scales and items in the HypoPT patients with reference to the active vitamin D compound administered (Fig. 2).

We did not detect any statistically significant group differences between the active vitamin D compounds administered in the HypoPT group (Fig. 2). A large variance is evident, however, which is indicative of the possibility of some dose‐dependent effects of the different vitamin D metabolites.

### The influence of combination therapy on scales and items of the HPQ 28

To analyze the influence of combination therapy in HypoPT patients with calcium, magnesium, and native vitamin D on the symptoms as given in the HPQ 28, we performed a group analysis of variance as well as a Spearman rank correlation (Appendix S2, Table B) of medication doses (magnesium, calcium, and native vitamin D, respectively) to scales and items of the HPQ 28.

**Fig 3:**
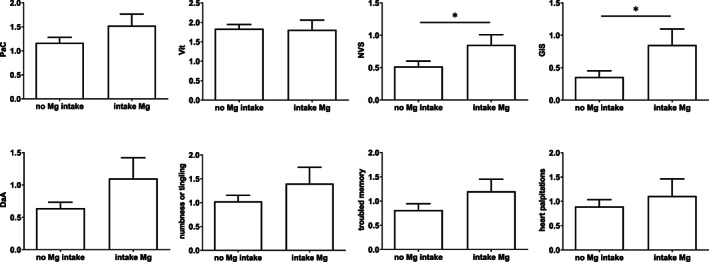
Influence of magnesium medication on scales and items of the HPQ 28 (*n* = 49). Data were analyzed using either Student's *t* test (normal distribution) or a Mann‐Whitney test (non‐normal distribution). *p value <0.05. PaC and Vit (six items; 4/294 items in 49 patients missing); GiS (two items; 1/98 items in 49 patients missing); DaA (five items; 1/245 items in 49 patients missing); NVS (five items; 4/245 items in 49 patients missing); one patient did not answer to item numbness and tingling and one did not answer to troubled memory. DaA = depression and anxiety; GiS = gastrointestinal symptoms; NVS = neurovegetative symptoms; PaC = pain and cramps; Vit = loss of vitality.

With respect to magnesium we grouped the patients into “no intake of magnesium” (*n* = 10) and “intake of magnesium” (*n* = 39). Patients treated with magnesium complained significantly more often on the scales GiS and NVS (*p* < 0.05, Fig. 3). The scale GiS reflects symptoms of abdominal pain and cramps, nausea, or upset stomach, whereas the scale NVS relates to symptoms such as trembling muscles, hot flushes or chills, weakness, dizziness, and diarrhea. No further significant group differences could be detected. The subsequent Spearman rank analysis (*r*_s_ = 0.29; *p* = 0.049) also revealed significant positive correlation between magnesium intake and the scale GiS. None of the other scales or items of the HPQ 28 correlated to the magnesium dose (Appendix S2, Table B).

**Fig 4:**
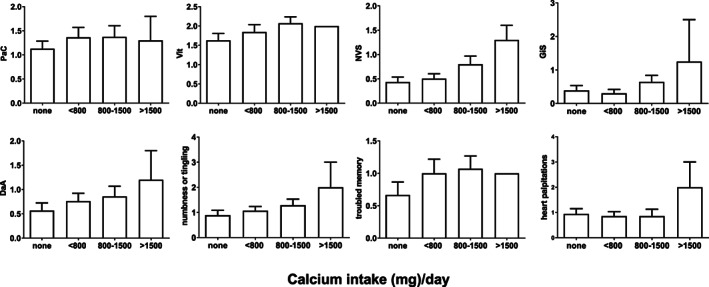
Influence of calcium medication on scales and items of the HPQ 28. Data were analyzed using either one‐way ANOVA (normal distribution) followed by a post‐hoc Bonferroni group comparison or a Kruskal‐Wallis test (non‐normal distribution) followed by a post‐hoc Dunn's group comparison. Groups: No calcium intake (*n* = 18); 800–1500 mg/d Mg (*n* = 15); >800 mg/d (*n* = 13); >1500 mg/d: (3375 and 4000 mg/d; *n* = 2). PaC and Vit (six items; 4/294 items in 49 patients missing); GiS (two items; 1/98 items in 49 patients missing); DaA (five items; 1/245 items in 49 patients missing); NVS (five items; 4/245 items in 49 patients missing); one patient did not answer to item numbness and tingling and one did not answer to troubled memory. DaA = depression and anxiety; GiS = gastrointestinal symptoms; NVS = neurovegetative symptoms; PaC = pain and cramps; Vit = loss of vitality.

Regarding calcium, patients were grouped into four groups “no intake” (*n* = 18), “below 800 mg/d” (*n* = 15), “800 to 1500 mg/d” (*n* = 14), and “above 1500 mg/d” (*n* = 2). Subsequent Spearman rank correlation analysis for calcium intake revealed significant correlation to the scale NVS (*p* = 0.044; *r*_s_ = 0.29) (Appendix S2, Table B). This reflects more complaints represented by NVS, such as trembling muscles, hot flushes or chills, weakness, dizziness and diarrhea with higher doses of calcium intake (Fig. 4).

Finally, with respect to vitamin D intake, three groups were formed: “no intake of native vitamin D” (*n* = 24), “approx. 1000–3000 IU vitamin D/d” (*n* = 19), and 10,000–40,000 IU vitamin D/d (*n* = 6). Group analysis of native‐vitamin D intake revealed no significant influence on any of the scales of the HPQ 28. Subsequent correlation analysis revealed no significant correlation (Appendix S2, Table B).

### Correlation of symptoms to the dose of active vitamin D

Table 3 lists the range of doses for the active vitamin D compounds calcitriol (*n* = 15), alfacalcidol (*n* = 22), and dihydrotachysterol (*n* = 6). Peak doses were 0.5 μg/d for calcitriol (43%) and 1 μg/d for alfacalcidol (38%).

**Table 3:** Dosage of Active Vitamin D Treatments in HypoPT Patients

Calcitriol		Alfacalcidol		DHT	
(μg/d)	*n*	(μg/d)	*n*	μg/d	*n*
0.13	1	0.5	3	130	1
0.25	1	0.75	3	270	1
0.50	7	1.00	8	600	1
0.75	3	1.25	3	1000	1
1	1	1.5	2	1500	2
1.25	1	2.0	3		
3.00	1				
Mean	*n* = 15	Mean	*n* = 22	Mean	*n* = 6
0.76 ± 0.68		1.11 ± 0.45		833 ± 597	

DHT = dihydrotachysterol; HypoPT = hypoparathyroidism..

Although we found that there were no differences between vitamin D treatment modalities and HPQ 28, there still may be some dose‐dependent effect of different active vitamin D compounds. To answer the question as to whether symptoms in HypoPT patients correlate to the dosage of the different active vitamin D compounds administered, we performed a Spearman rank correlation analysis (Table 4). The calcitriol dose correlated positively and significantly with three scales and one item (“pain and cramps”, “depression and anxiety”, “numbness and tingling”, and “heart palpitations”) with a rank correlation coefficient *r*_s_ of ~0.6.

**Table 4:** Correlation of Active Vitamin D Medication to Scales and Items of the HPQ 28

Active vitamin D compound	Correlation	PaC	Vit	GiS	DaA	NVS	Numbness and tingling	Troubled memory	Heart palpitations
Calcitriol (μg/d)	*r*_s_ ^a^	**0.567**	0.078	0.404	**0.643**	0.449	**0.582**	0.449	**0.604**
	*p* ^b^	**0.028**	0.784	0.135	**0.010**	0.093	**0.023**	0.094	**0.017**
	*n*	**15**	15	15	**15**	15	**15**	15	**15**
Alfacalcidol (μg/d)	*r*_s_	−0.027	0.056	0.109	0.122	0.012	−0.179	−0.061	−0.153
	*p*	0.905	0.804	0.629	0.590	0.959	0.438	0.788	0.509
	*n*	22	22	22	22	22	21	22	21
DHT (μg/d)	*r*_s_	0.377	−0.058	0.141	−0.574	0.265	0.045	−0.664	0.045
	*p*	0.461	0.913	0.790	0.234	0.612	0.933	0.150	0.933
	*n*	6	6	6	6	6	6	6	6

Significant correlations are marked bold.

DaA = depression and anxiety; DHT: dihydrotachysterol; GiS = gastrointestinal symptoms; HPQ 28 = 28‐item Hypoparathyroid Patient Questionnaire; NVS = neurovegetative symptoms; PaC = pain and cramps; Vit = loss of vitality.

^a^ Spearman rank correlation coefficient.

^b^ After Bonferroni correction *p* > 0.05 due to low number of patients and high number of parameters.

Linear regression analysis revealed a significant influence of calcitriol on the scales PaC (*r*_c_^2^ = 0.33; *p* = 0.017), DaA (*r*_c_^2^ = 0.25; *p* = 0.038), and the items “numbness and tingling” (*r*_c_^2^ = 0.51; *p* = 0.003) and “heart palpitations” (*r*_c_^2^ = 0.53; *p* = 0.002).

The calcitriol dose explains 33.0% of the variation of the scale PaC variation, 25% of the scale DaA, 51% of the items “numbness and tingling”, and 53% of the item “heart palpitations”. These are strong effects for every scale or item according to Cohen (0.6–1.1 = effect size).^(^ ^16^ ^)^

Alfacalcidol and dihydrotachysterol appear to have no detectable effect on any of the symptom scales (Table 4).

Principally, the possibility remains that different doses of calcitriol result in different levels of serum calcium, thus explaining the symptoms. To answer the question as to whether serum calcium levels influence the demonstrated effect of calcitriol on the four parameters, we employed a linear regression analysis model. The results are depicted in Table 5.

**Table 5:** Results of Regression Analysis of the Effect of Calcitriol and Calcium Serum Values on the Significant Scales and Items

Parameter	Coefficient	PaC	DaA	Numbness and tingling	Heart palpitations
Calcitriol	β	0.564	0.569	0.633	0.479
	*p*	0.042*	0.041*	0.021*	0.098
Serum calcium	β	0.188	0.170	0.054	0.032
	*p*	0.46	0.503	0.823	0.908

β = standardized coefficient parameter β; DaA = depression and anxiety; *p* = *p* value for single parameters; PaC = pain and cramps.

* *p* < 0.05 after correction for serum calcium.

The item “heart palpitations” was no longer significant after correction for serum calcium. However, correlation of the calcitriol dose with the scales PaC, DaA, and the item “numbness and tingling” remained significant after correction for serum calcium. This model therefore suggests that certain complaints are dependent on the dose of calcitriol but not on the dose of the other active vitamin D compounds. This effect was also independent of gender, age, underlying disease, kind of surgery, serum 25‐hydroxyvitamin D_3_, or phosphate values. Higher calcitriol doses had no effect on any of the laboratory values.

## Discussion

Lately, we developed the HPQ 28 questionnaire to characterize and quantify symptoms and complaints specifically of HypoPT patients.^(^ ^14^ ^)^ Scales and items of the HPQ 28 reflect different areas of complaints characteristic in patients with HypoPT and correlated to an extent with the laboratory parameters serum calcium, serum phosphate, and CPP.^(^ ^15^ ^)^ In this study, we investigated whether different treatment modalities influence symptoms and complaints in HypoPT patients.

Different options to treat HypoPT are available throughout the world. International therapy recommendations have been available since 2015,^(^ ^1^, ^2^ ^)^ which strongly advocate the use of activated vitamin D analogs in combination with calcium supplements. However, the recommendations do not specify which vitamin D analog is suggested. Table 6 depicts data concerning the pharmacokinetics of vitamin D analogs.^(^ ^1^, ^17^ ^)^ In Germany, all different vitamin D compounds have been approved for the treatment of HypoPT patients and are administered regularly.

**Table 6:** Different Active Vitamin D Compounds to Treat Chronic HypoPT^(^ ^1^ ^)^

Active vitamin D	Finally activated in	Time to onset (days)	Time to offset (days)	Typical dose (μg/d)
Calcitriol (1,25(OH)_2_D3)	Active	1–2	2–3	0.25–2.0
Alfacalcidol (1α(OH)D3)	Liver	1–2	5–7	0.5–4.0
Dihydrotachysterol (DHT, vitamin D2 derivative)	Liver	4–7	7–21	300–1000

HypoPT = hypoparathyroidism.

We detected no difference in serum calcium, serum phosphate, serum magnesium, CPP, calcium in 24‐hour urine, or 24‐hour phosphate excretion between the different active vitamin D compounds. However, more patients' parameters were in the reference ranges according to guidelines with calcitriol and DHT than with alfacalcidol.

Principally, the patients were well adjusted to the current guidelines. Eleven patients (73%) treated with calcitriol, three (50%) treated with DHT, 10 (48%) treated with alfacalcidol, and five (71%) not taking any active vitamin D compound were within the reference ranges of all six parameters. One has to take into account that not all data were available for every patient. Regarding the four serum parameters calcium, phosphate, CPP, and magnesium, 100% were in the reference range for calcitriol, 81% for alfacalcidol, and 83% for DHT. Comparing the achievement of the guidelines’ recommendations with the work by Meola and colleagues,^(^ ^6^ ^)^ we found in those patients (*n* = 24) with values for serum calcium, serum phosphate, CPP, and urinary calcium, 71% (*n* = 17) to be within the reference ranges. Our patients are therefore more controlled compared to the Italian study group, which reported that 34.1% met four targets (albumin adjusted serum calcium, phosphate, CPP, and 24‐hour urinary calcium).

Regarding symptoms and complaints measured by HPQ 28, there were no differences between the different active vitamin D compounds administered and those patients on other forms of therapy. However, the variance was very high; we therefore analyzed the influence of co‐medication with calcium, magnesium, and native vitamin D_3_ further.

The intake of magnesium supplements influenced two scales. In comparison with those not taking magnesium, patients complained more with respect to the scales NVS and GiS. There are items relating to gastrointestinal symptoms/complaints in both scales. For example, the item diarrhea belongs to the NVS scale and the item nausea to GiS. Because we know that the side effects of magnesium intake can include gastrointestinal symptoms,^(^ ^18^ ^)^ these results are in line with the everyday experience of clinical patient care. Hence, this finding confirms the usefulness of HPQ 28 to characterize patient complaints as specific symptoms.

When correlating calcium intake with HPQ 28 scales and items, we identified more complaints represented by the scale NVS, such as trembling muscles, hot flushes or chills, weakness, dizziness, and diarrhea as doses of calcium intake increased. The items “trembling muscles” and “weakness” correlated to calcium dose particularly, whereas the single item “diarrhea” did not correlate to calcium dose at all. On the one hand the symptoms we identified may be the reason for increasing calcium intake, or the consequence of high calcium intake on the other hand. We know from individuals taking calcium supplements that calcium medication can cause gastrointestinal (GI) symptoms such as constipation, excessive abdominal cramping, bloating, upper GI events, GI disease, GI symptoms, and severe diarrhea. These symptoms might be experienced by the patient as heart problems. This was suggested by a meta‐analysis demonstrating that an increase in the incidence of adverse GI events also increased the number of self‐reported myocardial infarctions in calcium‐treated patients but not controls.^(^ ^19^ ^)^ However, the item “heart palpitations” was not influenced by calcium intake in our patients. This may suggest, at least in part, that the symptoms and complaints are the reason for increasing calcium intake.

Although there were no differences between the effect of vitamin D compounds on HPQ 28 parameters, a high variance was evident. We were therefore interested in the effect of different doses of the individual vitamin D compounds. Although no dose effect was detectable in patients treated with alfacalcidol or DHT, in contrast, calcitriol dose effects were significant in three HPQ 28 scales when corrected for the effect of serum calcium. Summarizing these results, patients with higher doses of calcitriol suffer both greater depression and pain. The scale PaC includes the items pain in the (lower) back, joint pain or pain in the limbs, muscle pain, neck or shoulder pain, and muscle cramps. The items “joint pain or pain in the limbs”, “neck or shoulder pain” and most significantly “muscle cramps” were shown to correlate to calcitriol dosage. The scale DaA includes self‐blaming emotions, inner tension and restlessness, sorrowful thoughts, melancholia, and difficulty in making decisions. All single items that belong to the DaA scale except the item “difficulty to make decisions” were affected by the calcitriol dose significantly. However, these symptoms are not typical side effects of calcitriol. In the German national pharmaceutical register, known as the “Rote Liste,” the side effects of calcitriol are listed as anorexia, headache, nausea, skin erythema, and bladder infections, none of which appear in any of the significant scales PaC and DaA in HPQ 28. The dose‐dependent item “numbness and tingling” would suggest clinically that, in general, those with greater symptom load take more calcitriol. However, the dose of calcitriol is not determined according to symptoms but to attain and maintain the target range of serum calcium; the latter did not influence our results. This would suggest that the identified symptoms/complaints are independent of the actual calcium level determined. However, further analysis to identify other possible interfering parameters did not detect any influence of age, gender, underlying disease, or kind of surgery. Nevertheless, the number of patients is small and there remains the possibility that other, to date unidentified parameters influence our results.

It is of further interest that the other active vitamin D compounds did not demonstrate any dose effects influencing HPQ 28 scales and items. Reviewing the differences of the vitamin D compounds we studied (Table 6), calcitriol with the shortest half‐life could possibly result in more and/or greater fluctuations in serum calcium than the others tested; however, no data on this hypothesis exist. However, even fluctuating calcium levels probably do not explain the different pain locations as described by HPQ 28 in this study. These particular pain symptoms or complaints are not common for the majority of HypoPT patients and thus may prove to be a consequence of the therapy.

Hence, our data suggest that calcitriol induces dose‐dependent symptoms, in contrast to the other vitamin D compounds we studied.

A limitation of our study is the low number of study participants taking each individual vitamin D compound. A greater number of HypoPT patients would help to strengthen our results and thus conclusions. Patient numbers were too small to correct for all influencing parameters at the same time; the test could only be performed for one factor at a time. Comparison of those individual patients undergoing a change in their medication would be helpful toward future verification of any differential effects of the vitamin D compound actually implemented. Nonetheless, this might not be clinically feasible. Ultimately, this is the first study to our knowledge investigating laboratory values and symptoms dependent on available active vitamin D compounds.

In summary, we compared different treatment modalities in patients with HypoPT, by analyzing and evaluating laboratory values, and QoL according to symptoms and complaints. Although laboratory values in the majority of patients were in the reference ranges, patients reported a huge number of complaints. Using the recently developed HPQ 28 specific questionnaire, we identified effects of medication with calcium, magnesium, and calcitriol. Interestingly, serum values for all the patients treated with calcitriol were 100% in the reference range, although patients still demonstrated a number of dose‐dependent symptoms and complaints according to HPQ 28. This study presents the first data on specific complaints of HypoPT patients dependent on different treatment modalities. Our data would imply that in part the reduced QoL in these patients might be caused by one or a combination of the conventional treatment modalities. For the clinician treating patients with HypoPT, our data would suggest carefully considering patients' symptoms and complaints not only as caused by the disease itself but by the treatment.

## Conflict of Interest

During the last 3 years, BS has received lecture honoraria from Shire/Takeda, Novartis, Astra Zeneca, and Boehringer Ingelheim, and served as advisory board member of Shire/Takeda. CH‐L has received lecture honoraria from Heel, Servier, and Novartis, as well as royalties from Hogrefe Huber publishers. HS has served during the last 3 years as an advisory board member for Shire/Takeda, UCB, and Kyowa Kirin, and received speaker's fees from Shire/Takeda and Amgen. The other authors stated no conflict of interest.

### Peer review

The peer review history for this article is available at https://publons.com/publon/10.1002/jbm4.10586.

## Supporting information


**Appendix** **S1**: Supplement Table A

**Appendix** **S2**: Supplement Table B
