# Supplementary material for: The Influence of Conventional Treatment on Symptoms and Complaints in Patients With Chronic Postsurgical Hypoparathyroidism
Source: JBMR Plus. 2022 Feb 1;6(2):e10586. doi: 10.1002/jbm4.10586 (PMC8861984; doi:10.1002/jbm4.10586)
Supplement: Supplementary file 1 — Appendix S1: Supplement Table A [file JBM4-6-e10586-s002.docx]

Supplement A

| **Magnesium** | | **Calcium** | | **native Vit D** | | **Thiazide** | |
| --- | --- | --- | --- | --- | --- | --- | --- |
| (mg/d) | n | (mg/d) | n | IU/d | n | (mg/d) | n |
| 0 | 39 | 0 | 18 | 0 | 24 | 0 | 42 |
| 120 | 2 | 200 | 1 | 143 | 1 | 12.5 | 1 |
| 150 | 1 | 500 | 9 | 400 | 2 | 25 | 5 |
| 200 | 1 | 600 | 4 | 800 | 1 | 37.5 | 1 |
| 300 | 4 | 750 | 1 | 1000 | 3 |  |  |
| 600 | 1 | 950 | 1 | 1429 | 2 |  |  |
| 900 | 1 | 1000 | 10 | 1800 | 1 |  |  |
|  |  | 1200 | 2 | 2000 | 1 |  |  |
|  |  | 1500 | 1 | 2200 | 1 |  |  |
|  |  | 3375 | 1 | 2309 | 1 |  |  |
|  |  | 4000 | 1 | 2500 | 1 |  |  |
|  |  |  |  | 2857 | 4 |  |  |
|  |  |  |  | 3658 | 1 |  |  |
|  |  |  |  | 10,000 | 1 |  |  |
|  |  |  |  | 20,000 | 4 |  |  |
|  |  |  |  | 40,000 | 1 |  |  |

d= day; n= number of patients
