# Supplementary material for: The Influence of Conventional Treatment on Symptoms and Complaints in Patients With Chronic Postsurgical Hypoparathyroidism
Source: JBMR Plus. 2022 Feb 1;6(2):e10586. doi: 10.1002/jbm4.10586 (PMC8861984; doi:10.1002/jbm4.10586)
Supplement: Supplementary file 2 — Appendix S2: Supplement Table B [file JBM4-6-e10586-s001.docx]

Supplement B

| **Parameter** | **Spearman rho**  **correlation** | **PaC** | **Loss of**  **Vit** | **GiS** | **DaA** | **NVS** | **Numbness and tingling** | **Troubled memory** | **Heart palpitations** |
| --- | --- | --- | --- | --- | --- | --- | --- | --- | --- |
| **Mg dose/d (mg)** | r_s_^a^ | .93 | -.06 | **.286** | .147 | .265 | .127 | .213 | .073 |
|  | p-value^b^ | .190 | .968 | **.049** | .318 | .069 | .394 | .147 | .628 |
|  | n^c^ | 48 | 48 | **48** | 48 | 48 | 47 | 48 | 47 |
| **Ca dose/d**  **(mg)** | rs | .096 | .209 | .132 | .213 | **.289** | .202 | .232 | .013 |
|  | p-value | .513 | .149 | .365 | .143 | **.044** | .168 | .109 | ·930 |
|  | n | 49 | 49 | 49 | 49 | **49** | 48 | 49 | 48 |
| **Native Vit D suppl·/d (IU)** | r_s_ | -.108 | -.078 | -.061 | .012 | -.186 | .015 | -.087 | -.073 |
|  | p-value | .462 | .594 | .677 | .934 | .202 | .917 | .553 | .620 |
|  | n | 49 | 49 | 49 | 49 | 49 | 48 | 49 | 48 |

d=per day; IE=international units; PaC=pain and cramps; vit=vitality; GiS=gastrointestinal symptoms; DaA= depression and anxiety; NVS=neurovegetative symptoms
